# Theorizing "cultural signatures" in worldwide personal networks.

**DOI:** 10.12688/openreseurope.23361.3

**Published:** 2026-07-31

**Authors:** José Luis Molina, Alejandro Dinkelberg, Miroslav Pulgar, Christopher McCarty, Miguel Ángel González-Casado, Ángel Sánchez

**Affiliations:** 1 Antropologia social i cultural, Universitat Autonoma de Barcelona Departament d'Antropologia Social i Cultural, Bellaterra, Catalonia, 08193, Spain; 2 Matemáticas, Universidad Carlos III de Madrid Departamento de Matematicas, Leganés, Community of Madrid, 28903, Spain; 3 Anthropology, University of Florida, Gainesville, Florida, FL 32611, USA

**Keywords:** culture-structure, personal networks, cultural institutions, kinship.

## Abstract

**Background:**

This paper advances the theory of “cultural signatures” in personal networks by integrating Goodenough’s concept of propriospect—the individual’s internalized model of culture—with a personal network approach to social structure. We define cultural signatures as the structural traces left by cultural institutions—such as associations, religious practices, or educational organizations—in personal networks. To illustrate this theoretical framework, we revisit Fredrik Barth’s ethnography of Sohar, an Omani town characterized by multiculturality and gender segregation. Using the propriospect approach, we show how shared cultural codes of politeness and self-restraint sustain social diversity and how institutions (market, mosques, Wali court, and neighborhoods) shape the gendered topology of personal networks.

**Methods:**

To test the theoretical framework, we conducted a web survey in six countries selected across Gelfand’s tight–loose cultural range, collecting 1,815 personal networks, about 300 by country, with egos nominating 30 alters and the alter-alter ties. Along with this rich structural information, participants provided sociodemographic data and information about their moral values. The data was analyzed using forest tree methods to predict the country of origin from structural, sociodemographic, or moral values variables.

**Results:**

We demonstrate that network structures, especially cohesion measures, significantly contribute to predicting respondents’ cultural background (country of origin in this case), supporting the hypothesis that social interaction patterns encode identifiable cultural imprints.

**Conclusions:**

By linking cognitive models of culture with measurable meso-level social structures, the paper provides a conceptual and methodological framework for analyzing how cultural diversity manifests in patterns of social interaction.

## Introduction

The classic debate between “culture” and “social structure” has often led to confusion over multiple definitions and levels of analysis of both concepts ( Eisenstadt, 1986, 1989; Molina et al., 2025). In this work, we aim to contribute to this debate by adopting Goodenough’s proposal of individual “propriospect” as the working operationalization of “culture” ( 1973) and by conceiving personal networks as samples of “social structures”. This approach enables us to study the interaction between these two dimensions of social life (i.e., the ensemble of values, norms, beliefs, and the patterns of social interaction) in a consistent manner. It also enables us to theorize about the social mechanisms that may explain their mutual influence. In particular, we are interested in explaining why and how we can find traces of personal network structures left by cultural institutions, a phenomenon that we have dubbed “cultural signatures” ( Molina et al., 2022).

To address this goal, we first introduce the concept of “cultural signatures”. Next, we will introduce the concept of *propriospect* proposed by Goodenough to conceptualize terms such as “national cultures”, “ethnolinguistic groups”, and “subcultures”. Using the concept of propriospect, we will illustrate how cultural diversity at the country level can be understood as a combination of widely shared institutions and specific cultural traditions at the individual level. Once we understand how to conceptualize the complexity of cultural components, we will study an ethnographic case to apply these ideas to patterns of social interaction (i.e., personal networks) in a multiethnic enclave, specifically the town of Sohar in Oman ( Barth, 1983). By analyzing ethnographic data from Oman, we will gain insights into how structure and composition of personal networks reflect these cultural variations, especially regarding gender segregation. After developing our theoretical framework, we will present the methods and data from a survey conducted by the authors across six countries to test and interpret the results. Finally, we will outline our conclusions and the limitations of our work.

## Cultural signatures

We introduced the concept of “cultural signatures,” building on the idea of “social signatures” proposed by Saramaki et al. (2014), which suggests that it is possible to identify a unique pattern of time allocation to communication with alters by each ego (its “signature”). Other authors have confirmed this finding in multichannel communications and noted its persistence over time ( Heydari et al., 2018; Li et al., 2018; Martin et al., 2024). In our case, drawing on a rich dataset of personal networks of 472 migrants from Africa and Latin America to the United States and Spain ( Molina et al., 2022), we were able to predict with different methods the country of origin and religion (a proxy of cultural traits), looking at the ensemble of structural measures derived from the alter-alter matrix of personal networks (a proxy of social structures). Notice that both “social” and “cultural” signatures are measured at the individual level, looking at the pattern of social relationships around the ego. We interpreted this finding as cultural institutions (i.e., kinship and the gender/sex system, religious cults, education, political organization, and so on) leaving traces in personal network structures (the “cultural signature”). However, the actual social mechanisms that may explain this process were not theoretically elaborated. In addition, the question remained whether this phenomenon could be found in the general population or just in migrant groups. To properly address these open issues, we need to present first the concept of “propriospect”.

## The Propriospect approach

In *Culture, Language, and Society*, Ward H. Goodenough draws on a classic ethnographic description of the Vaupés people, located in the northwest Amazon region along the Vaupés River, which crosses the borders of Colombia and Brazil. In this region, about 10,000 indigenous people share similar ways of livelihood, kinship, and religious rituals. They all participate in a network of exogamic marriage exchanges that bind patrilineal clans together. Following the patrilocal rule, adult males, their wives, and children reside in a local long-house village, where the wives come from other clans and places. What is remarkable about this case is that these indigenous people speak 20 mutually unintelligible languages, such that a man must marry a woman who speaks a different language. Every language is associated with a tribe composed of various clans that serve as both political and ceremonial units.

Each village is, by definition, multilingual, with women speaking their own tribal languages and children speaking both their father’s and mother’s languages. The local language must be used in the presence of the males of the village. Additionally, Tukano, the language of the most important Vaupés tribe, serves as a *lingua franca* in the region.

This example enables Goodenough to reflect critically on the common-sense notion that distinct languages differentiate distinct peoples, and by extension, that different peoples have different cultures. His aim is to address multilingualism and multiculturalism as a departure point, as is often the case in urban societies. To achieve this task, he poses the “propriospect” concept.

A propriospect is an individual’s internalized model of the world, a structured and partial version of the cultural environment, as much as speakers have their own *idiolects* of a given language. It includes all that a person knows, believes, feels, and values, along with the expectations that orient their behavior. Unlike idiolects, the concept of propriospect implies competence in different cultures, associated with different sets of others. Quoting Goodenough (p. 99):

This concept may be represented as:

p=(a+b+c+…)+x

in which the individual’s propriospect ( *p*) consists of the several cultures ( *a,b,c,* etc.) he attributes to corresponding sets of people, together with other forms, beliefs, values, and recipes ( *x*) as he has developed from his experience of things apart from other people, and that he attributes to no other person.

This competence in different sets of standards attributed to sets of others is distinct from membership or identification with a given group, although it may be the case. Different groupings of people, naturally associated with shared activities (such as those developed by occupations, clubs, associations, and the like), may develop distinct sets of standards, traditions, or subcultures in much the same way languages develop dialects. In this way, the Culture (with capital C) of a given society is equal to the set of different subcultures or traditions performed by its members. In the following example, individual 1 is competent in the subcultures or traditions *a*, *b*, and *d* of Culture *A*, and also is competent in Culture *K* (which has its own traditions), whereas individual 2 is competent in not exactly the same traditions of Culture *A* and that of individual 1, but is competent in another Culture *L.* Additionally, there may be individuals who are solely familiar with the traditions associated with Culture *A*, which in this example may be considered the dominant culture in this society:

$$p_{1}=\left(A_{1:}\;a_{1},b_{1},--,d_{1},--,\ldots \right)+\left(K_{1},--,--,\;\ldots \right)+x_{1}$$

$$p_{2}=\left(A_{2:}\;a_{2},b_{2},c_{2},--,--,\;\ldots \right)+{\;}_{--,}\;L_{2},--,\;\ldots )+x_{2}$$

$$p_{i}=\left(A_{i:}\;a_{i},b_{i},--,--,\;j_{i}\right)+\left(--,--,\;J_{i}\right)+x_{i}\;\text{where}\;J\;\mathrm{can}\;\mathrm{be}\;\text{null}.$$

This conception of Culture as the sum of individual propriospects helps us to conceptualize “national cultures”, “subcultures”, and “ethnolinguistic groups” easily. When we refer to the “culture” of a given country, we are actually referring to the set of traditions, institutions, and customs available to all of its members. A given sample of individuals from that country will share a subset of these traditions, but not necessarily all of them. Furthermore, in some societies, men and women may hold different gender subcultures. Similarly, minorities and ethnolinguistic groups can have their own distinct culture (referred to as *K* and *L* in the example) while also sharing traditions from the dominant culture *A.* Thus, the propriospect approach offers a realistic conceptualization of culture in contemporary multicultural societies, understood as a dynamic and complex system able to reproduce core traditions shared by most of its members, while allowing a wide range of internal and external diversity.

The next section presents a practical application of this concept to the conceptualization of the social life of a culturally diverse town in Oman, reflecting on the connections between subcultures and patterns of interaction.

## Personal networks in a multiethnic enclave

### The ethnographic site

Fredrik Barth, in his book *Sohar: Culture and Society in an* *Omani Town* ( 1983) describes the life of a multiethnic city, seeking the mechanisms that explain the reproduction of its cultural diversity.

Sohar is a historically significant port situated on the northern coast of Oman, in the Arabian Peninsula. As a traditional Islamic city, it is administratively divided into wards, with the central one containing the two essential pillars of urban society: the market ( *suq*) and the fortress ( *hosn*), which serves as a center for law and order. While the market is a self-organized institution with three subsidiary sites, the fish market, the meat and vegetable market, and the largely covered main bazaar, the Sultan’s appointed governor (the Wali) holds court and controls all public matters related to the town with its own guard.

The town’s population consists of Hindus, Muslims (with distinct factions), Baluchis from Persia, Sindhis from Pakistan, and the Kutchi from Gujarat (Western India), speaking their own languages, apart from the Bedouin tribes, which are bound by patrilineal organization, among others. Some ethnic groups may be overrepresented in certain occupations or levels of wealth and status, but there is no clear segregation in this regard.

The clientele of these places is almost exclusively masculine. Women, from all ethnic groups, live separated from public arenas and avoid contact with unrelated males. They live almost exclusively in the neighborhood and their compounds, and even in these circumstances, keep their faces covered with a *burqa*, often wrapped in a cloak ( *abba*), when moving through back streets away from home.

What is remarkable here is that, in daily life and within the confines of each gender, people from different ethnic groups and categories mix and meet, develop friendships, attend wedding celebrations, and, in the case of males, form business partnerships that unite individuals from diverse ethnic affiliations, languages, and faiths. Women also frequently gather to visit each other, often over coffee, or exchange informal conversations. The apparent differences in ethnicity, status, and faith are managed through the “common ideals of poise, grace, and politeness” (p. 7), which help to mute these differences and sustain faithful relationships over time.

With the aid of the propriospect approach, we can represent the cultural diversity of the Sohar town as follows:

$$p_{i\;\text{male}}=\left(A_{i:}\;a_{i},b_{i},--,--,\;j_{i}\right)+\left(--,--,\;J_{i}\right)+x_{i}\;$$

$$p_{i\;\text{female}}=\left(A_{i:}\;a_{i}--,c_{i},--,\;j_{i}\right)+\left(--,--,\;J_{i}\right)+x_{i}\;$$

being *a*, the shared set of values and norms that allows the reproduction of cultural differences, in this case, the etiquette ethos, in which both genders are competent, and *b* and *c *, the corresponding gender subcultures. To this matrix, it is possible to add occupational subcultures, common religion, and so on, apart from being competent in the other cultural traditions from Pakistan, Iran, or India.

### The personal networks of Sohar people

How do personal networks of Sohar people reflect these propriospects? According to Barth’s qualitative account, men in Sohar gather and establish relationships in various public and semi-public settings or foci ( Feld, 1981), whereas women primarily interact within the confines of their neighborhoods.

The market, with more than 1,000 people at any given time and 200 shops, is the preferred place for approaching a man, even by the local authorities. Social interactions are welcome, open, and continuous. A second focus of activity is attending one of the many mosques scattered throughout the city, especially on Fridays, although the conversation is not as free and casual as it is in the market. The third place of importance is the Wali’s court, which attracts considerable numbers of people waiting for a reception or a decision on their demands. Socialization in this situation is possible but limited to the people who have business there. Apart from these main foci, men can affiliate with a club created by the government, which receives a small subsidy, and organize recreational and cultural activities. In general, attendance at any meeting is small, and just the elected members spend most of their free afternoons working there. Finally, men can gather in cafés or tea shops, meet acquaintances on the way to the orchards, or pay visits to family members.

Barth introduces the example of Ali, who is Arab and claims to have 20 friends, aside from his family members. These friends are similar in age, ranging from 25 to 27 years old, and share occupations in governmental or municipal services, as well as in business firms. However, they differ in terms of ethnicity—comprising Arabs, Baluchis, and Ajam—and religion, including both Sunni and Shiah Muslims. Aside from four friends he has known since childhood, the rest of his friends come from the diverse backgrounds mentioned above.

The case of Ali contrasts with that of males from other minority groups, such as the Zidgalis, among others, who often have strong kinship obligations or specific calendars or rituals that reinforce their ties to the boundaries of their own ethnic group.

For women, sexual segregation and restricted interaction limit their social networks beyond family members and neighbors. They are generally not allowed to attend mosques, clubs, or cafes. Their social contact is largely confined to infrequent visits to markets with trusted male escorts, short trips along the nearest streets from their home, and interactions in public spaces like hospitals. These few foci limit the opportunities to establish new social contacts. At the local level, women often belong to a social circle of 6 to 8 close neighbors along with their children, which may include daily visits or multiple visits each day. These groups of women tend to be more diverse than those of men. The members can vary not only in terms of ethnicity and religious background but also in wealth, age, and ex-slave background —a category that would hinder heterogeneous male friendships.

After analyzing the social worlds of men and women in Sohar, the first apparent trait is the heterogeneity in composition (ethnicity and religion), especially concerning women, who naturally mix people from all conditions in their domestic spheres. This heterogeneity is possible thanks to the elaborated code of politeness and contention that characterizes life in Soar (the component *a* in the propriospect).

The second trait concerns gender segregation ( *b* and *c*, respectively), which results in distinct interaction patterns for men and women. Men tend to have a larger and more dispersed network of friends and acquaintances, which allows them greater freedom in choosing their relationships. In contrast, women typically form a smaller and denser network of close neighbors, primarily based on propinquity. While both men and women maintain kinship ties, men tend to have a wider circle of friends, whereas women often cultivate a tighter circle of close connections.

This general pattern may differ for ethnic minorities, particularly those based on kinship or those with elaborate rituals, which can reduce heterogeneity and increase the density of network ties (see Molina et al., 2025 for a discussion).

What is important here is that, drawing on the concept of propriospect, which suggests that different traditions and a set of shared activities give rise to distinct network structures (in terms of size, density, clustering, and so on), we can meaningfully infer gender and ethnicity from personal network configurations. Nevertheless, this translation of cultural institutions into personal network structures is not direct, but approximate, as we will see in the 6-country study presented below; a question that requires theoretical elaboration.

## Theorizing cultural signatures in personal networks

The concept of propriospect and its translation to personal network structures contributes to closing the gap posed by many authors ( Nadel, 1957; Radcliffe-Brown, 1940; Sorokin & Richard, 2017) that culture and social structures are separate domains of social life that should be approached with different epistemological strategies. Instead, this exercise presents a theoretical model that explains the presence of cultural signatures in both migrant and general populations, and opens up further avenues of research.

The first proposition of our theoretical framework is that “culture”, understood here as the complex of traditions and memberships of people, influences observed patterns of social interaction. In fact, research on the relationship between culture and networks, despite the different approaches and acknowledgment of complex interactions, suggests precisely that the dominant direction is *from* culture *to* networks. The expressions “Culture in Networks” ( McLean, 2016), “Networks from culture” ( Fuhse, 2021, 2009; Fuhse & Gondal, 2022), “Cultural Worldviews influence Network Composition” ( Vaisey & Lizardo, 2010), or “Cultural Tastes Shape Personal Networks” ( Lizardo, 2006) are examples of this. To be more precise, McLean poses that cultural narratives and repertoires shape social relationships, Fuhse convincingly shows how shared meanings and identities generate network structures, and guide network development, Vaisey & Lizardo offer empirical evidence of how cultural worldviews influence the selection of social contacts and Lizardo arguments that cultural tastes produce homophily and structure personal networks.

Once acknowledged the general precedence of culture to networks, we suggest using the concept of “cultural institution” instead of “cultural tradition” or “subculture” as the component of the propriospect vector because it allows to distinguish between traditions associated with membership (i.e., with cohesive patterns of interaction) from traditions in general (shared by disconnected people). In this vein, following Valdés del Toro and Valdés Gázquez (1989) we can conceive “institutions” as a three-component device that evolves over time: a set of values, a set of behaviors, and a set of actors. When the relationship between two or three of these sets is weak, we can refer to them as “defective institutions,” such as individual worldviews, a common set of behaviors (e.g., etiquette and self-restraint in our example), or the women who occasionally greet their neighbors. These institutions may leave identifiable traces in personal networks, but they are not necessarily distinctive. Conversely, when the three sets are strongly connected, we can speak of “corporative institutions” where values, behaviors, and actors are clearly identifiable, such as occupational guilds, churches, associations, and formal or informal organizations. Notice that the use of “cultural institutions” ( Martin, 2009) emphasizes the concepts of regularity, repetition, shared values, and expected norms of behavior for different roles, as well as the existence of specific actors. If these corporative institutions are present in a given propriospect, they will leave identifiable structures in personal networks, like clusters of alters with specific characteristics.

Actually, the “foci of interaction” ( Feld, 1981; Small, 2009) are instances of cultural institutions, which provide the contexts of focused interactions and the performance of roles (classrooms with students and professors, churches with priests and parishioners, job places with organizational charts, and so on). In our example the market (men) or the neighborhood (women) would be instances of corporative institutions that leave clearly identifiable structures in terms of size, composition, and density, among other network characteristics.

In sum, from our theoretical framework we would expect that people within a given country will share a set of common cultural institutions despite their internal diversity. Among these *shared* cultural institutions, we expect that the *corporative* ones (those that connect shared values, behaviours and membership) will leave identifiable traces in personal networks structures, specifically, in cohesive network measures. These patterns or “cultural signatures” should be present in the set of individual propriospects, and should contribute to predict the country of origin more accurately.

## Six-country survey on personal networks

As we said, to test whether it was possible to find cultural signatures in the general population beyond migrant groups, we conducted a 6-country survey that collected 1,815 valid personal networks with a fixed number of alters (30) and the alter-alter ties ( González-Casado et al., 2026), along with some questions about social values and ethnolinguistic affiliation. The results confirm that personal network structures (i.e, the pattern of connections among alters) play a role in predicting the country (a proxy of “culture”). To describe how these structural components are implemented into our model, we now turn to the study’s data and methods.

## Data

The data form part of a larger ongoing data collection process within the MapCDPerNets project ^1^, in which we collect data on online and offline personal networks across nine countries. For this article, we use data from the first six countries ^2^ where collection is already completed. The countries selected follow the diversity index elaborated by Gelfand et al. (2011) along a dimension of “tight” and “loose” world cultures, determined by the strength of social norms and the tolerability for deviation from those. In all six countries, participants aged 18 years and older were recruited to complete an online Qualtrics survey after providing informed consent online. These samples are not nationally representative, but they enable us to capture cross-cultural variation in personal network structures. The country of origin serves as a proxy for the cultural influences that individuals are experiencing and determines where the country is positioned on the tight-looseness scale.

For each country, the target sample size was 300 valid responses. The surveys were run between January and November 2025. Due to the complexity of the approach and the project’s international scope, we utilized a combination of self-service platforms and full-service research agencies, including TGM Research (France, Morocco, Sweden), Prolific (Mexico), ISRA (Romania), and Clickworker (Romania, South Africa). After data collection and cleaning, a total of 1,815 valid responses were retained for analysis (see Table 1). Participants received compensation of approximately $5–$8 per completed survey, varying by panel provider and country. The median completion time was approximately 40 minutes, underscoring the task’s complexity.

**Table 1.** Summary of survey participants for the six countries (France, Morocco, Mexico, Romania, South Africa, and Sweden).

Country	Female	Male	Other	Prefer not to say	Total (valid responses)	Total responses
France	197	138	0	0	335	431
Morocco	116	186	0	0	302	374
Mexico	123	163	2	2	290	319
Romania	180	138	0	0	318	383
South Africa	194	95	0	0	289	307
Sweden	113	165	3	0	281	334
**Total**	**923**	**885**	**5**	**2**	**1,815**	**2,148**

Our survey (see Supplementary Material) comprises multiple sections that capture sociodemographic data, social norms and personal networks. The participants provide information on age, gender, marital status and household size and indicate in three items the perception of social norms in their country taken from the National Study of Youth and Religion, the European Values Survey, and Gelfand et al., 2011 respectively. The survey’s central component is the elicitation of the participants’ personal networks. Following González-Casado et al. (2026), using a fixed-number name generator, participants are first asked to list exactly 30 alters (people nominated by each respondent). They then provide information on each alter’s characteristics (name interpreter) and determine the personal network structure by indicating whether two alters would interact with each other in the participant’s absence (name interconnector). The fixed network size facilitates comparability and standardisation while allowing variability in network structures.

Table 1 provides details on the survey participants for each country, as well as the total number of valid responses. In France, Romania, and South Africa, the majority of respondents are female, whereas in Morocco, Sweden, and Mexico, male participants predominate. The column “Total (valid responses)” indicates the number of valid responses after data cleaning has been applied. Across countries, sample sizes range from 284 to 335 participants, which aligns with the initial target sample size of 300 participants.

## The random forest classification method

Our analytical approach builds on the framework of Molina et al. (2022), utilizing a larger dataset from the general population and a broader spectrum of personal network metrics to link network structures with cultural signatures. Molina et al. (2022) examined the predictability of origin countries from migrants based on six network metrics and evaluated three statistical approaches, such as the Random Forest method, to assess the information density in personal network structures.

Here, we focus on the Random Forest method ( Breiman, 2001) for single-label classification to predict participants’ country of origin by integrating personal network metrics as predictors. In this way, we can test whether we can find “signatures” of the national cultures in the patterns of social relationships. This approach captures non-linear relationships and handles heterogeneous feature types ( Parmar et al. 2019). Following González-Casado et al. (2024), we compute 40 personal network metrics to define personal network structures and subsequently apply Principal Components Analysis (PCA) ( Jolliffe 2016) as a dimension-reduction technique to avoid data redundancies. The PCA identifies a low-dimensional factor representation of each personal network that explains most of the variability in their metrics. Thus, the 40 metrics are reduced to six principal components (PCs) that serve as input for our prediction model. As suggested by our theoretical framework, structural information about the personal networks might contain clues about the person’s cultural environment (here: country).

We enhance the classification by incorporating additional variables that capture the emotional closeness between alter and ego, ego characteristics, and social norms and their perception (see Supplementary Material). The survey captures the emotional closeness on a 5-point Likert scale between the ego and the alters. From this assessment of emotional closeness, we derive the *μ factor* ( Tamarit et al. 2022), which describes the distribution of social circles within the personal network. It summarizes the number of people at each level of closeness in a single value.

Apart from these structural measures, we also draw on survey items that characterize the ego (age group, gender, household size, marital status). Finally, we incorporated three indicators of social norms, constructed from various items, that capture a) the individuals’ moral cosmopolitanism orientation, b) a classification of cultural schemas across countries (using Correlational Class Analysis, see Boutyline 2017) and c) how closely each respondent aligns with the overall cultural model (derived from Cultural Consensus Analysis, see Romney et al. 1986). In particular, to capture the moral orientation of each participant we used the types proposed by Vaisey and Lizardo (2010): expressivist, utilitarian, theistic, communitarian, or relativist; to assign the cultural schemas about social norms obtained via Correlational Class Analysis from the norms items from Gelfand et al. (2011) we classified respondents in one of the three resulting classes; and, finally, to assess the level of cultural consensus, we produced an individual index with Cultural Consensus Analysis, an inverse factor analysis that estimates simultaneously the consensus model and each respondent’s deviation.

Next, we test several combinations of features to optimize our Random Forest classifier and determine which yields the best performance. We compare our model results with those following the classification framework presented by Molina et al. (2022). Specifically, we evaluate our model performance against the same three (dummy) predictors: (1) a uniform dummy that assigns all classes (here: countries) equal probability; (2) a stratified dummy that assigns probabilities based on class distribution, and (3) a most-frequent dummy, which always predicts the most common country. The roughly balanced distribution of the classes yields similar performance across all three dummy classifiers.

In the following, we examine a series of configurations to classify the ego’s country of origin based on information about their personal network structure and ego characteristics. Nevertheless, we present only the optimised model with the optimal set of features in detail. The extended analysis and a detailed description of the optimisation process are provided in the Supplementary Material.

## Results and discussion

The observed performance of the Random Forest Classifier describes the amount of information that is present within a set of features for predicting the country of origin of participants from six distinct countries. The best model maximises the information input while performing better than other sets of features or a dummy classifier. After data cleaning and splitting the dataset into training and test subsets, the model is initialized with several sets of features to optimize the classifier.

All model configurations are evaluated based on their prediction accuracy in the test subsets, considering test accuracy, balanced accuracy, and F1-Score (see Table 2; see also Supplementary Material). Test accuracy indicates the relative number of correct predictions; balanced accuracy introduces an equal weight to each class, and F1-Score summarises the model’s correct predictions (precision), relative to the overall number of possible correct predictions (recall). All three metrics result in an identical ranking of the model configurations.

**Table 2.** Random Forest Classifier configurations with six distinct parameter sets.

Model Configuration	# Features	Test accuracy	Test balanced accuracy	Test macro F1	Accuracy over dummy
Molina et al. (2022) — selected network features incl. μ	6	0.245	0.242	0.227	+49%
Structural features (PCs only)	6	0.242	0.243	0.227	+48%
Structural features PCs + μ only	7	0.256	0.255	0.229	+56%
Demographics/social norms (EXTRA_COLUMNS only)	8	0.442	0.445	0.426	+170%
PCs + extra features (NO μ)	13	0.451	0.449	0.439	+175%
**PCs + extra features (+ μ)**	**14**	**0.493**	**0.493**	**0.477**	**+200%**

In line with Molina et al. (2022), we initially select an equivalent feature set for evaluating prediction performance. This model performs substantially better than the dummy predictors and shows equal improvements in accuracy as the model configuration only based on structural features (PCs only). The configuration of structural features including the descriptor of the social circles (μ) shows a similar effect. The largest improvement in accuracy shows adding the demographic and social norm features. We evaluate three feature sets that result in a similar performance range by achieving nearly up to three times the accuracy than the most frequent dummy predictor: (1) a model that only relies on the sociodemographic data without any structural information (+170%); (2) a model based on structural, social norms and demographic data (+175%) and (3) a model including all available features, including the distribution of the alters in social circles of closeness (+200%). Hence, we have three models that are performing qualitatively similar.

The classification model leads to the best possible accuracy and integrates all fourteen features: six principal components, four demographic variables, three variables related to social norms and the descriptor of distributed alters in the social circles. The Random Forest classifier achieves a test accuracy of 0.493 here, which is nearly three times higher than the best dummy predictor (0.163). This suggests that the features add substantial informational value to the model, enabling it to distinguish between countries (see also the Summary Table in the Supplementary Material).

Overall, the model performance improves as additional features are introduced. Although adding social and demographic features yields significant improvements, the structural features nonetheless contribute complementary information that further enhances the model’s predictive accuracy.

Table 3 lists the model performance across each category (country). The F1-Scores vary from 0.42 (SW) to 0.60 (MO). They facilitate identifying which country’s personal networks follow more distinct patterns and allow, therefore, a better prediction. The prediction performances vary notably between countries, which suggest various levels of distinct features or a high variability between the participants within a country.

**Table 3.** Model performance across countries (PCS + extra features).

*Country*	*Precision*	*Recall*	*F1-score*	*Support*
FR	0.406	0.641	0.497	64
MO	0.489	0.780	0.601	59
MX	0.600	0.569	0.584	58
RO	0.609	0.215	0.318	65
ZA	0.692	0.327	0.444	55
SW	0.411	0.426	0.418	54
Accuracy	–	–	0.493	355
Macro avg	0.535	0.493	0.477	355
Weighted avg	0.534	0.493	0.476	355

Random Forest Classifiers enable the estimation of each feature’s influence on the classification task, and these feature contributions to the model’s prediction performance are assessed using SHAP values. The mean absolute SHAP value of each input feature estimates its relative influence on the classification task, where higher values indicate a stronger influence.

**Figure 1.**
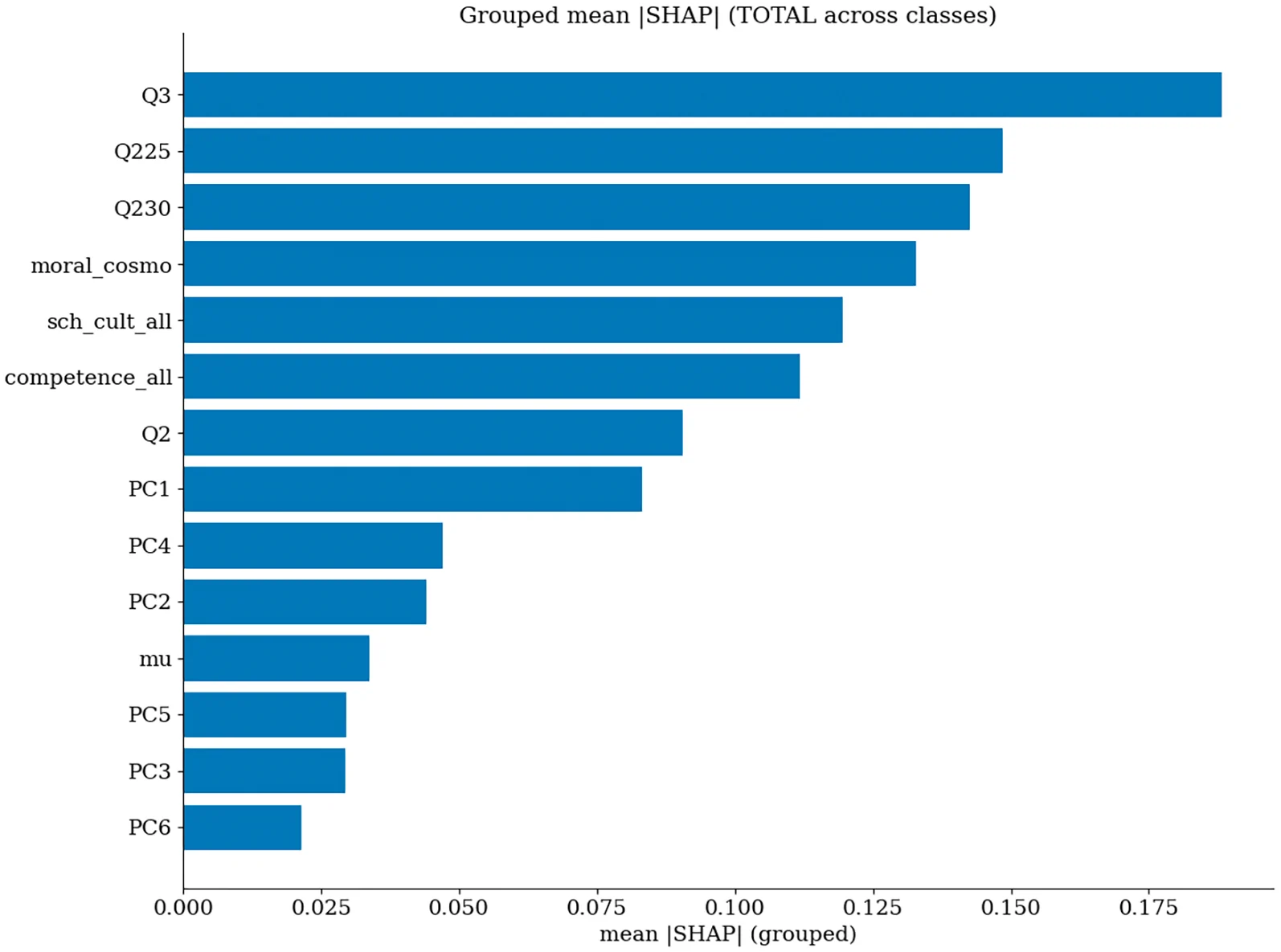
Mean absolute SHAP values for feature importance across all countries. Legend: Qi = sociodemographic questions; moral_cosmo = value orientation; competence_all = consensus analysis model; sch_cult_all = class of cultural schema, and PC i = structural measures of personal networks, with cohesion measures represented by PC1.

**Figure 2.**
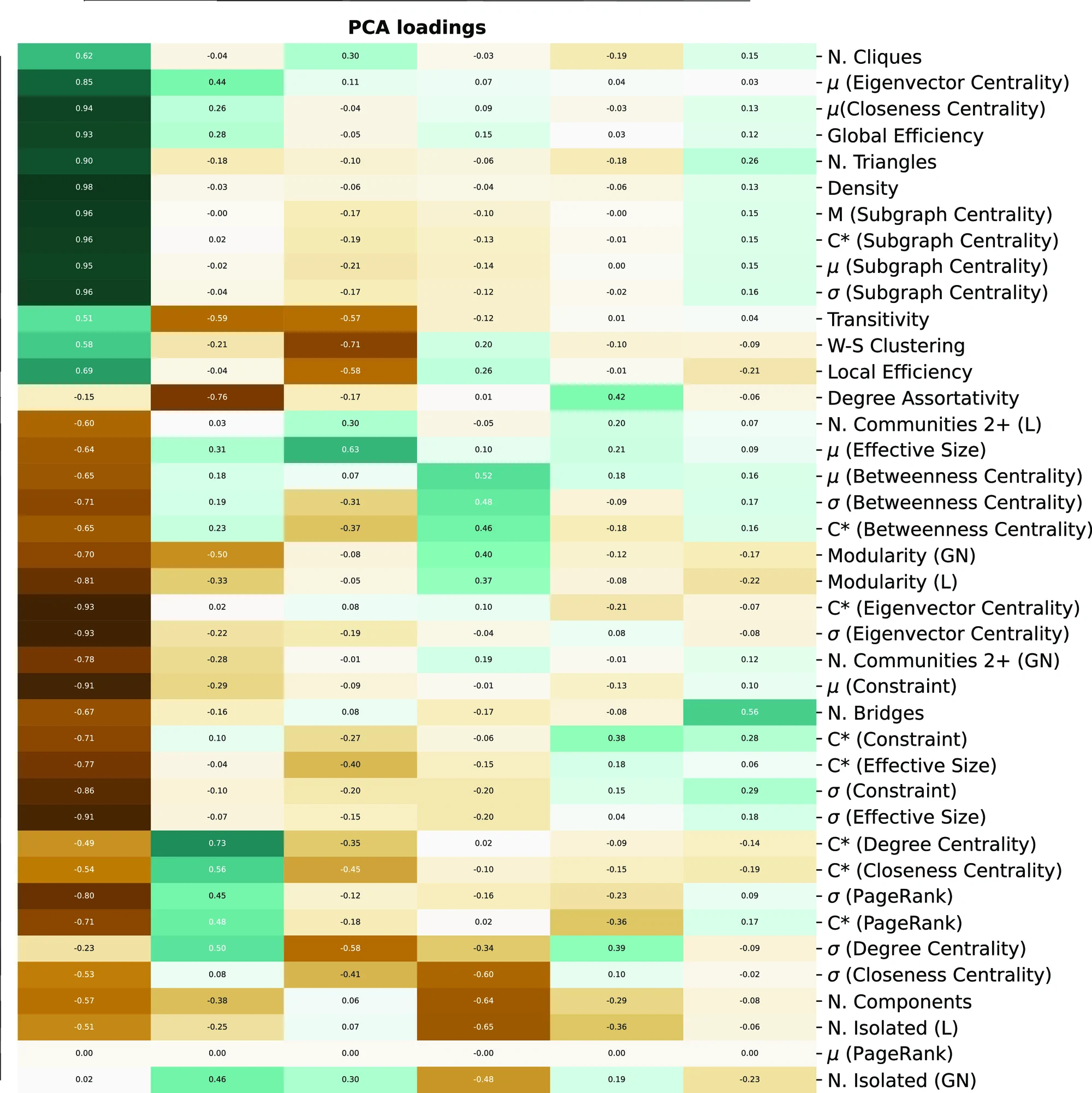
Principal component loadings from network measures. Legend: The columns, starting from the left and colored by intensity, indicate PC1, PC2 – PC6. Cohesive network measures such as density, number of cliques, and number of triangles exhibit a strong positive association with PC1, whereas modular fragmentation measures are negatively associated with PC1.

The SHAP analysis in Figure 1 compares the influence of each feature on the prediction performance for the country of origin and reveals a strong influence of the demographic features, such as the ego’s age (Q3) and marital status (Q230). Furthermore, social norm variables are less influential but also contribute significantly to the prediction performance, representing the common understanding of aspects of social life within each country. While PC1 still shows a substantial contribution (0.1), most structural features have only a minimal influence on the prediction outcome. Critically, PC1 measures *cohesive networks*, tightly knitted, with short paths between the alters due to high efficiency and high subgraph centrality, as shown in Figure 2. Conversely, the rest of PCs measure centralized, fragmented, modular, assortative and bridged networks respectively. Finally, the influence of the descriptor of the social circles *(μ*) is similarly limited.

In sum, the personal network structures across countries contain traces of cultural signatures, particularly the cohesion measures identified by PC1, that enhance the classification of the countries of origin of egos. This finding aligns with our hypotheses that corporative institutions (those that include membership along with a set of values and behaviours) leave identifiable traces on personal network structures. In addition, it is worth noting that the PCs as a whole contain a set of features that summarise distinct aspects of personal network structures. Consequently, when we consider their aggregated influence (sum of SHAP values) as a group, it exceeds that of any individual feature contribution.

## Conclusions and limitations

This paper proposes a theoretical and methodological framework for identifying cultural signatures in personal network data, narrowing the traditional divide between cultural and social structure approaches. To achieve this goal, it was necessary to compare levels of analysis in both domains to be consistent, i.e., Goodenough’s concept of propriospect, which captures individual adaptation of shared traditions, and personal networks with a large number of alters, which capture not just the individual agency but the set of institutions in which the ego is affiliated.

The Random Forest analysis of six countries confirms that personal network structural measures significantly contribute to predicting respondents’ cultural background, specifically the country of origin in this case. However, it also shows that there is no direct correspondence of cultural institutions and identifiable network structures. To address this situation, we re-examined Barth’s ethnographic case of Sohar, a multicultural site, and we illustrated how gender segregation and corporative institutions, such as markets and neighborhoods, produce distinct relational topologies.

The theoretical model developed here advances two propositions: first, building on extant research, that culture influences both the content and the patterning of social relations, and second, that only some cultural components—particularly corporative institutions—translate into identifiable and cohesive network structures. This complex translation of propriospect’s institutions to personal network configurations may explain why the strong predictive capacity of structural measures can be enhanced with other individual measurements.

Beyond anthropology, we think that this approach has implications for cross-cultural psychology, sociology, and computational social science, providing a scalable method for studying cultural diversity and social cohesion in multicultural settings.

Nonetheless, several limitations must be acknowledged. First, the six-country survey is exploratory and not nationally representative. Moreover, the Random Forest classifier, although robust, remains a statistical approximation of complex social processes. In this regard, we aim to further develop the propriospect concept using the available data on tastes and individual preferences collected alongside personal networks. Furthermore, we recognize the need to develop a more culturally sensitive set of network measures that simultaneously capture both compositional and structural dimensions. Last but not least, cross-country differences in recruitment platforms and sample composition may affect the classification results.

A significant portion of the planet’s population is currently sharing their personal connections with international tech companies on a daily basis. For good or ill, the concept of cultural signatures opens an avenue of research into predicting human cognition and behavior across its cultural diversity.

## Ethics and consent

This project was approved by the Committee of Ethics in Research of Universidad Carlos III de Madrid (CEI23_10_SANCHEZ_Anxo). Participants provided consent before participating in the web survey.

## AI tools

The authors used Consensus to check references, and Grammarly to check English style.

## Data Availability

The dataset is available on Zenodo at https://doi.org/10.5281/zenodo.20701232, Creative Commons Attribution 4.0 International.

## Footnotes

^1^
Project details under https://mapcdpernets.es/

^2^
France, Morocco, Sweden, Mexico, Romania and South Africa
