# Supplementary material for: Theorizing "cultural signatures" in worldwide personal networks
Source: Open Res Eur. 2026 Jul 31;6:115. Originally published 2026 Apr 16. [Version 3] doi: 10.12688/openreseurope.23361.3 (PMC13555058; doi:10.12688/openreseurope.23361.3)
Supplement: Peer review history

# Reviewer report: Reviewer response for version 3

**DOI:** 10.21956/openreseurope.26742.r78370

**Published:** 2026-08-06

**Authors:** Gabriel Otero

**Affiliations:** 1 Universidad Diego Portales, Santiago, Chile

I am satisfied with this revised version. The authors have addressed all of my previous comments carefully, and I have no further substantive concerns. I believe the manuscript is now suitable for indexing.

If applicable, is the statistical analysis and its interpretation appropriate?

Yes

Is the study design appropriate and is the work technically sound?

Yes

Is the work clearly and accurately presented and does it engage with the current literature?

Yes

Are the conclusions drawn adequately supported by the results?

Partly

Are sufficient details of methods and analysis provided to allow replication by others?

Yes

Are all the source data and materials underlying the results available?

Yes

Reviewer Expertise:

class, personal networks, social cohesion

I confirm that I have read this submission and believe that I have an appropriate level of expertise to confirm that it is of an acceptable scientific standard.

# Reviewer report: Reviewer response for version 2

**DOI:** 10.21956/openreseurope.26372.r77412

**Published:** 2026-07-22

**Authors:** Isidro Maya-Jariego

**Affiliations:** 1 Universidad de Sevilla, Sevilla, Spain

The authors have made improvements to the manuscript, clarifying several elements. The revised version has improved the manuscript's internal consistency. The discussion of national culture and institutions is primarily interpretive, offering theoretical insights rather than direct empirical evidence. As such, the paper provides a thought-provoking theoretical contribution that could serve as a basis for future empirical research.

If applicable, is the statistical analysis and its interpretation appropriate?

Yes

Is the study design appropriate and is the work technically sound?

Yes

Is the work clearly and accurately presented and does it engage with the current literature?

Yes

Are the conclusions drawn adequately supported by the results?

Partly

Are sufficient details of methods and analysis provided to allow replication by others?

Yes

Are all the source data and materials underlying the results available?

Yes

Reviewer Expertise:

Social Psychology

I confirm that I have read this submission and believe that I have an appropriate level of expertise to confirm that it is of an acceptable scientific standard.

# Reviewer report: Reviewer response for version 2

**DOI:** 10.21956/openreseurope.26372.r77411

**Published:** 2026-07-21

**Authors:** Gabriel Otero

**Affiliations:** 1 Universidad Diego Portales, Santiago, Chile

I thank the authors for their careful and constructive response. The revision represents a genuine improvement over Version 1, particularly in its structural reorganization and theoretical elaboration. My remaining substantive request is an explicit acknowledgment in the limitations section of the potential confounding effects of panel heterogeneity and sample composition (point 5). Thus, I am comfortable moving from “approved with reservations” to “approved” once the proposed limitations sentence and the remaining textual errors are addressed, as none of these issues requires new analysis or further review. My detailed comments are provided below.

 1. Social vs. cultural signatures distinction and novelty relative to Molina et al. (2022). The added passage clarifying that both concepts are “measured at the individual level, looking at the pattern of social relationships around the ego” is helpful, and the explicit statement of the paper's two open questions (the absence of theorized mechanisms in the 2022 paper, and whether the phenomenon extends beyond migrant populations) now makes the contribution much clearer. I note, however, that the clarification sidesteps rather than fully resolves the conceptual asymmetry originally raised (also by Reviewer 1). In fact, social signatures concern intra-individual stability, cultural signatures concern trans-individual patterning. The added sentence establishes a common measurement level but not a common analytical logic. I consider this acceptable for the paper's purposes, since the new theoretical section carries the explanatory weight, but a single additional sentence acknowledging this asymmetry would strengthen the framing.

 2. The underspecified mechanism linking propriospect to network structure, and the late/underdeveloped treatment of defective vs. corporative institutions. This was my central concern, and it has been substantially addressed. The new section “Theorizing cultural signatures in personal networks”, now placed before the empirical study, transforms the paper's logic from post-hoc interpretation to hypothesis-driven testing. Crucially, the theory now generates a falsifiable expectation: corporative institutions (linking values, behaviors, and membership) should leave traces specifically in cohesive network measures. The subsequent finding that PC1, now explicitly identified as capturing cohesion (high efficiency, high subgraph centrality, short paths), is the most influential structural predictor provides a meaningful theory-evidence connection that was absent in v1.

 3. Selective engagement with the culture-networks literature. The added sentence summarizing the specific contributions of McLean, Fuhse, Vaisey & Lizardo, and Lizardo is thin, but given the format constraints of the article I consider it sufficient. However, this added passage contains minor errors that must be corrected: "Fushe" should read "Fuhse" and "desconnected" should read "disconnected".

 4. The Sohar section as illustrative narrative rather than formal analysis. The authors' rationale, that Barth's ethnography contains no quantitative relational data and that the section's purpose is to illustrate intra-group propriospect variation under extreme gender segregation, is reasonable, and the relocated theoretical section now does the analytical work I was asking the Sohar case to perform.

 5. Sample representativeness and panel-specific biases. I appreciate the acknowledgment in the response letter that nationally representative multi-country network samples are beyond the reach of any single project. However, the substantive concern was not representativeness per se but differential selection bias. Since gender (Q2) is itself a predictor in the model, sample-composition differences could inflate the demographic features' contribution. I do not require new analyses, but the limitations section should explicitly acknowledge that cross-country differences in recruitment platform and sample composition constitute a potential confound for the classification results. This is one or two sentences and should be added.

 6. PCA interpretability in the main text. The substantive interpretation of PC1 as cohesion, and the brief characterization of the remaining PCs (centralization, fragmentation, modularity, assortativity, bridging), now appear in the main text. The proportion of variance explained by the six components is still relegated to the Supplementary Material; I would prefer a single sentence reporting it in the main text, but this is now a minor point.

 7. “Cultural signatures” as interpretation vs. demonstration. The restructured paper now presents the cultural-institutional interpretation as a theoretically derived hypothesis tested against a specific prediction (cohesion measures). The deeper issue, that country of origin remains a coarse proxy conflating cultural, economic, and demographic variation, persists, but the authors acknowledge it and the framing is now defensible.

 8. The discussion remains somewhat compressed, but the integration problem identified in v1 is resolved.

 Minor issues

 The numerical inconsistency between the text ("test accuracy of 0.476") and Table 2/Table 3 (0.493 for the full model) — carried over from v1 — must be reconciled or explained (I suspect 0.476 refers to the weighted F1 in Table 3, in which case the text should say so). The reference list still contains a duplicate entry for Barth (1983). Additional typos: "orign," "instituions," "propiospect" (twice), "Soar" (for Sohar), and "networ ks" in the SHAP paragraph.

If applicable, is the statistical analysis and its interpretation appropriate?

Yes

Is the study design appropriate and is the work technically sound?

Yes

Is the work clearly and accurately presented and does it engage with the current literature?

Yes

Are the conclusions drawn adequately supported by the results?

Partly

Are sufficient details of methods and analysis provided to allow replication by others?

Yes

Are all the source data and materials underlying the results available?

Yes

Reviewer Expertise:

class, personal networks, social cohesion

I confirm that I have read this submission and believe that I have an appropriate level of expertise to confirm that it is of an acceptable scientific standard.

# Reviewer report: Reviewer response for version 1

**DOI:** 10.21956/openreseurope.25274.r72718

**Published:** 2026-06-03

**Authors:** Gabriel Otero

**Affiliations:** 1 Universidad Diego Portales, Santiago, Chile

This paper addresses the relationship between culture and social structure as observed through personal network data and makes a good effort to bridge cognitive anthropology (Goodenough's propriospect) with quantitative network analysis. The paper is very interesting, but several structural and argumentative gaps need to be addressed before the work can be considered fully convincing.

**Introduction**

 - The concept of "cultural signatures”, defined as structural traces of cultural institutions in personal networks, is introduced with reasonable clarity and connected to the prior literature on "social signatures" (Saramaki et al., 2014). However, the conceptual distinction between social and cultural signatures deserves more rigorous treatment here rather than being deferred implicitly.

 - Moreover, the introduction would benefit from a sharper statement of what is novel relative to Molina et al. (2022), since the current paper appears to extend that prior work to a general population.

**Theoretical framework**

 - The choice of Goodenough's propriospect as an operationalization of "culture" is well-motivated. Also, the application to the Vaupés ethnographic case is effective as a useful illustration of the concept's flexibility. That said, some issues limit the section's theoretical payoff. First, the connection between propriospect and the structural properties of personal networks is asserted rather than fully theorized. The paper states that "culture influences observed content and patterns of interactions", but the mechanism remains underspecified. Thus, the authors could further elaborate on that issue. Second, the distinction between "defective institutions" and "corporative institutions" is introduced at a late stage and feels somewhat underdeveloped. Third, the paper's engagement with the broader culture-networks literature is somewhat selective. Works like Lizardo (2006), Vaisey and Lizardo (2010), and McLean (2016) are cited but not discussed.

**Ethnographic case: Sohar**

 - The Barth (1983) material on Sohar is well-chosen as an illustration of how propriospect components, specifically the shared etiquette ethos and gendered subcultures, map onto distinct personal network topologies. However, this section functions primarily as an illustrative narrative rather than as a formal case analysis. The propriospect formulas applied to Sohar are presented with notation that implies analytical precision, but the mapping from the ethnographic description to these formal representations is not made explicit.

**Data and Methods**

 - The sample representativeness issue is acknowledged in a single brief sentence ("not nationally representative, strictly speaking") but deserves more careful treatment. The use of Prolific, TGM, ISRA, and Clickworker panels introduces platform-specific selection biases that differ by country, potentially confounding cross-national comparisons.

 - The reduction of 40 network metrics to 6 principal components via PCA is methodologically defensible, but the paper does not report what proportion of variance these components explain, nor does it provide enough information to assess what substantive network properties each PC captures. This information is apparently in the Supplementary Material, but some of it should appear in the main text to make the analysis interpretable.

**Results**

 - The main finding, that network structures contribute predictive information about country of origin beyond what demographics and social norms alone provide, is very interesting. However, the interpretation of this result as evidence of "cultural signatures" is inferential rather than demonstrated. What has been empirically shown is that personal network features predict country of origin. Calling this a "cultural signature" involves the additional theoretical claim that country of origin functions as a valid proxy for cultural influence, and that the network-level predictability stems from cultural institutions specifically rather than from other country-level factors. 

 - The transition from the results section to the propriospect discussion is somewhat abrupt and creates a structural discontinuity that is confusing for the reader. The results are presented, and then the paper goes to the ethnographic and theoretical material without a clear discussion section that integrates them.

**Conclusions and limitations**

 - The conclusions are appropriately cautious in some respects. However, the final paragraph gesturing toward implications for "predicting human cognition and behavior" across cultural diversity is somewhat speculative.

 - The paper would benefit from a more detailed discussion that explicitly connects the empirical results, the Sohar illustration, and the propriospect framework.

If applicable, is the statistical analysis and its interpretation appropriate?

Yes

Is the study design appropriate and is the work technically sound?

Yes

Is the work clearly and accurately presented and does it engage with the current literature?

Yes

Are the conclusions drawn adequately supported by the results?

Partly

Are sufficient details of methods and analysis provided to allow replication by others?

Yes

Are all the source data and materials underlying the results available?

Yes

Reviewer Expertise:

class, personal networks, social cohesion

I confirm that I have read this submission and believe that I have an appropriate level of expertise to confirm that it is of an acceptable scientific standard, however I have significant reservations, as outlined above.

# Response

**Published:** 2026-06-16

**Authors:** José Luis Molina

**Affiliations:** Antropologia social i cultural, Universitat Autonoma de Barcelona Departament d'Antropologia Social i Cultural, Bellaterra, Catalonia, Spain

Reviewer #2 We sincerely thank the reviewers for their insightful comments and criticisms. We have implemented most of the suggestions and we have restructured the paper to make it clearer and more understandable. All concern expressed by the reviewers have been addressed separately.  

 #1. The concept of “cultural signatures”, defined as structural traces of cultural institutions in personal networks, is introduced with reasonable clarity and connected to the prior literature on “social signatures” (Saramaki et al., 2014). However, the conceptual distinction between social and cultural signatures deserves more rigorous treatment here rather than being deferred implicitly. Moreover, the introduction would benefit from a sharper statement of what is novel relative to Molina et al. (2022), since the current paper appears to extend that prior work to a general population. Correct. We introduced this text to emphasize that both social and cultural signatures are measurements at the ego level, and highlight the novel paper contributions: “We introduced the concept of “cultural signatures,” building on the idea of “social signatures” proposed by Saramaki et al. (2014), which suggests that it is possible to identify a unique pattern of time allocation to communication with alters by each ego (its “signature”). Other authors have confirmed this finding in multichannel communications and noted its persistence over time (Heydari et al., 2018; Li et al., 2018; Martin et al., 2024). In our case, drawing on a rich dataset of personal networks of 472 migrants from Africa and Latin America to the United States and Spain (Molina et al., 2022), we were able to predict with different methods the country of origin and religion (a proxy of cultural traits), looking at the ensemble of structural measures derived from the alter-alter matrix of personal networks (a proxy of social structures). Notice that both “social” and “cultural” signatures are measured at the individual level, looking at the pattern of social relationships around the ego. We interpreted this finding as cultural institutions (i.e., kinship and the gender/sex system, religious cults, education, political organization, and so on) leaving traces in personal network structures (the “cultural signature”). However, the actual social mechanisms that may explain this process were not theoretically elaborated. In addition, the question remained whether this phenomenon could be found in the general population or just in migrant groups. To properly address these open issues we need to present first the concept of “propriospect”.

 #2. The choice of Goodenough’s propriospect as an operationalization of “culture” is well-motivated. Also, the application to the Vaupés ethnographic case is effective as a useful illustration of the concept’s flexibility. That said, some issues limit the section’s theoretical payoff. First, the connection between propriospect and the structural properties of personal networks is asserted rather than fully theorized. The paper states that “culture influences observed content and patterns of interactions”, but the mechanism remains underspecified. Thus, the authors could further elaborate on that issue. Second, the distinction between “defective institutions” and “corporative institutions” is introduced at a late stage and feels somewhat underdeveloped. The reviewer is right that the concept appears later in the paper. Now we have added a theoretical section before the presentation of the empirical study, and elaborated the concept as follows: “Once acknowledged the general precedence of culture to networks, we need to define more precisely what do we understand by “culture”. Within the propriospect approach, we suggest using the concept of “cultural institution” instead of “cultural tradition” or “subculture” because it allows to distinguished traditions associated with membership from traditions in general. In this vein, following Valdés and Valdés (1989) we can conceive “institutions” as a three-component device that evolves over time: a set of values, a set of behaviors, and a set of actors. When the relationship between two or three of these sets is weak, we can refer to them as “defective institutions,” such as individual worldviews, a common set of behaviors (e.g., etiquette and self-restraint in our example), or the group of women who occasionally visit their neighbors. These institutions may leave identifiable traces in personal networks, but they are not necessarily distinctive. Conversely, when the three sets are strongly connected, we can speak of “corporative institutions” where values, behaviors, and actors are clearly identifiable, such as occupational guilds, churches, associations, and formal or informal organizations. If these corporative institutions are present in a given propriospect, they will leave identifiable structures in personal networks, like clusters of alters with specific characteristics. Note that the concept of “foci of interaction” (Feld, 1981; Small, 2009) is in fact a result of the existence of cultural institutions, which provides the contexts of focused interactions and the performance of roles (classrooms with students and professors, churches with priests and parishioners, job places with organizational chats, and so on). In our example the market (men) or the neighborhood (women) would be instances of corporative institutions that leave clearly identifiable structures in terms of size, composition, and density, among other network characteristics. In this regard, the use of “cultural institutions” (Martin, 2009) emphasizes the concepts of regularity, repetition, shared values, and expected norms of behavior for different roles, as well as the existence of specific actors.” In addition, we clarify that PC1 refers to cohesion network measures, as anticipated in the theoretical framework, in which corporate institutions, those that imply membership and mutual connections, leave identifiable traces on personal network structures.

 #3. Third, the paper’s engagement with the broader culture-networks literature is somewhat selective. Works like Lizardo (2006), Vaisey and Lizardo (2010), and McLean (2016) are cited but not discussed. Given the space available we added the last phrase to the paragrah. “The first proposition of our theoretical framework is that “culture”, understood here as the complex of traditions and memberships of people, influences observed patterns of social interaction. In fact, research on the relationship between culture and networks, despite the different approaches and acknowledgment of complex interactions, suggests precisely that the dominant direction is from culture to networks. The expressions “Culture in Networks” (McLean, 2016), “Networks from culture” (Fuhse, 2021; 2009; Fuhse & Gondal, 2022), “Cultural Worldviews influence Network Composition” (Vaisey & Lizardo, 2010), or “Cultural Tastes Shape Personal Networks” (Lizardo, 2006) are examples of this. To be more precise, McLean poses that cultural narratives and repertoires shape social relationships, Fushe convincingly shows how shared meanings and identities generate network structures, and guide network development, Vaisey & Lizardo offer empirical evidence of how cultural worldviews influence the selection of social contacts and Lizardo arguments that cultural tastes produce homophily and structure personal networks.”

 #4. The Barth (1983) material on Sohar is well-chosen as an illustration of how propriospect components, specifically the shared etiquette ethos and gendered subcultures, map onto distinct personal network topologies. However, this section functions primarily as an illustrative narrative rather than as a formal case analysis. The propriospect formulas applied to Sohar are presented with notation that implies analytical precision, but the mapping from the ethnographic description to these formal representations is not made explicit. The ethnograhy does not include quantitative information on social relations, apart from some basic figures. Nevertheless, the aim of this section is to illustrate with an extreme case of gender segregation how propriospects do vary at the individual level, even within the same religious or ethnic group. The new theoretical section elaborates on the mechanisms that may explain the results. #4.  The sample representativeness issue is acknowledged in a single brief sentence (“not nationally representative, strictly speaking”) but deserves more careful treatment. The use of Prolific, TGM, ISRA, and Clickworker panels introduces platform-specific selection biases that differ by country, potentially confounding cross-national comparisons. Yes, the reviewer is right. We removed the expression “strictly speaking”. The complexity of collecting representative samples at the national level cannot be addressed by a single project, not even by an international organization. In fact, our contribution is to provide about 300 cases older than 18 years old by country, with a large number of alters. To our knowledge, this is the first time this type of data, with all its limitations, is available.    

 #5. The reduction of 40 network metrics to 6 principal components via PCA is methodologically defensible, but the paper does not report what proportion of variance these components explain, nor does it provide enough information to assess what substantive network properties each PC captures. This information is apparently in the Supplementary Material, but some of it should appear in the main text to make the analysis interpretable. Yes, indeed. We added to the Supplementary Material the details of the network measures that are relevant for each one of the PCs. As we said before, the PC1, the more relevant, includes cohesion measures.

 #6. The main finding, that network structures contribute predictive information about country of origin beyond what demographics and social norms alone provide, is very interesting. However, the interpretation of this result as evidence of “cultural signatures” is inferential rather than demonstrated. What has been empirically shown is that personal network features predict country of origin. Calling this a “cultural signature” involves the additional theoretical claim that country of origin functions as a valid proxy for cultural influence, and that the network-level predictability stems from cultural institutions specifically rather than from other country-level factors.  The reviewer is right. We think that the new theoretical section justifies better our interpretation of the results (see #2).  

 #7. The transition from the results section to the propriospect discussion is somewhat abrupt and creates a structural discontinuity that is confusing for the reader. The results are presented, and then the paper goes to the ethnographic and theoretical material without a clear discussion section that integrates them. Yes, this problem is solved in the new version.

 #8. The conclusions are appropriately cautious in some respects. However, the final paragraph gesturing toward implications for “predicting human cognition and behavior” across cultural diversity is somewhat speculative.   Yes, it is. But, given its placement at the end of the paper as a suggestive idea that deserves further research, we think it is justified.  

 #9. The paper would benefit from a more detailed discussion that explicitly connects the empirical results, the Sohar illustration, and the propriospect framework. Yes, it is true. On our opinion, the new version addresses all these concerns satisfactorily.

# Reviewer report: Reviewer response for version 1

**DOI:** 10.21956/openreseurope.25274.r72716

**Published:** 2026-04-23

**Authors:** Isidro Maya-Jariego

**Affiliations:** 1 Universidad de Sevilla, Sevilla, Spain

The article is well-written and, using personal network data, it illustrates the interdependence of culture and structure.

 The parallel between "cultural signatures" and "social signatures" is complex. The concept of social signatures seems to refer to (intra)individual stability in the allocation of time within a set of alters. The concept of cultural signatures, on the other hand, seems to refer to trans-individual stability in the allocation of time within a set of alters (which the authors attribute to national culture). If the former focuses on individual differences, the latter focuses on consistency within subgroups. Furthermore, there may potentially be many factors that explain membership in a cultural subgroup or a national culture, which are crucial for understanding inter-subject similarities within categories.

 It is also stated that "The country of origin serves as a proxy for the cultural influences that individuals are experiencing and determines where the country is positioned on the tight-looseness scale." However, individuals of the same nationality can vary widely within the same culture. For example, it could happen that individuals from the Andean region of Argentina share more similarities with the border region of Bolivia than with Buenos Aires, despite being from the same country (or the same "national culture"). It might be useful to have some measurement of the degree of cultural commitment of each individual to connect "culture" with the structural properties of their personal networks. It would be helpful to clarify whether culture is "measured" or simply inferrered by belonging to a particular country.

 In this sense, it is stated that "the personal network structures across countries contain traces of cultural signatures that enhance the classification of the countries of origin of egos." However, if I understand the models presented correctly, it seems that what is being shown is that personal networks have explanatory value regarding the country of origin, even considering the variability of social norms and other demographic aspects in the sample. Is stating that these are "cultural signatures" an interpretation by the authors or a direct description of the results?

 Likewise, the conclusions state that "personal network structural measures significantly contribute to predicting respondents' cultural background, specifically the country of origin in this case." However, isn't the ability to predict country of origin the only thing that has been empirically verified?

 After the Results section, there is a jump to the "propriospect approach" that is difficult for the reader to connect to the preceding analysis. Does this correspond to the Discussion of the previous results? Or is the intention to connect different case studies? It would be beneficial to improve the integration of the different sections that make up the text.

 Minor corrections:

 - In the list "that capture the individuals’ moral cosmopolitanism orientation, b a classification of cultural schemas (...)" I think adding a), b), c) would improve readability.

 - It seems that something is missing in this sentence: "(1) a model that only relies on the without any structural information (+170%);"

 - It would be helpful to put the direct quote from Goodenough in quotation marks to indicate where the quote begins and ends.

 - Review: "Following Valdés and Valdés (1989)". It seems is only Valdés.

 - Correct: "2025for a discussion"

 - Please check if the following text in the reference list is correct: "Omani Town: Fredrik Barth, in his book Sohar. Culture and Society in an Omani Town, describes the life of a multiethnic city, seeking the mechanisms that explain the reproduction of its cultural diversity; 1983."

If applicable, is the statistical analysis and its interpretation appropriate?

Yes

Is the study design appropriate and is the work technically sound?

Yes

Is the work clearly and accurately presented and does it engage with the current literature?

Yes

Are the conclusions drawn adequately supported by the results?

Partly

Are sufficient details of methods and analysis provided to allow replication by others?

Yes

Are all the source data and materials underlying the results available?

Yes

Reviewer Expertise:

Social Psychology

I confirm that I have read this submission and believe that I have an appropriate level of expertise to confirm that it is of an acceptable scientific standard, however I have significant reservations, as outlined above.

# Response

**Published:** 2026-06-16

**Authors:** José Luis Molina

**Affiliations:** Antropologia social i cultural, Universitat Autonoma de Barcelona Departament d'Antropologia Social i Cultural, Bellaterra, Catalonia, Spain

We sincerely thank the reviewers for their insightful comments and criticisms. We have implemented most of the suggestions and we have restructured the paper to make it clearer and more understandable. All concern expressed by the reviewers have been addressed separately.

   #1. The parallel between “cultural signatures” and “social signatures” is complex. The concept of social signatures seems to refer to (intra)individual stability in the allocation of time within a set of alters. The concept of cultural signatures, on the other hand, seems to refer to trans-individual stability in the allocation of time within a set of alters (which the authors attribute to national culture). If the former focuses on individual differences, the latter focuses on consistency within subgroups. Furthermore, there may potentially be many factors that explain membership in a cultural subgroup or a national culture, which are crucial for understanding inter-subject similarities within categories. Thanks for pointing this out. We think that drawing on the “social signature” concept for building the new one is justified because, in both cases, we measure the pattern of the ego’s ties across a collection of personal networks. The interpretation of the findings as traces of cultural institutions is, in our opinion, now better theoretically justified, addressing issues of country-level cultural variation with the “propriospect” approach, along with a clearer explanation of the concepts of corporate and defective cultural institutions.  In addition, we added this phrase at the end of the first paragraph in the “Cultural signatures” section: “Notice that both “social” and “cultural” signatures are measured at the individual level, looking at the pattern of social relationships around the ego. We interpreted this finding as cultural institutions (i.e., kinship and the gender/sex system, religious cults, education, political organization, and so on) leaving traces in personal network structures (the “cultural signature”). However, the actual social mechanisms that may explain this process were not theoretically elaborated. In addition, the question remained whether this phenomenon could be found in the general population or just in migrant groups.”

 #2. It is also stated that “The country of origin serves as a proxy for the cultural influences that individuals are experiencing and determines where the country is positioned on the tight-looseness scale.” However, individuals of the same nationality can vary widely within the same culture. For example, it could happen that individuals from the Andean region of Argentina share more similarities with the border region of Bolivia than with Buenos Aires, despite being from the same country (or the same “national culture”). It might be useful to have some measurement of the degree of cultural commitment of each individual to connect “culture” with the structural properties of their personal networks. It would be helpful to clarify whether culture is “measured” or simply inferrered by belonging to a particular country. Yes, this point was not well addressed in our previous version. We added this paragraph to the introductory section to make our aims explicit: “Using the concept of propriospect, we will illustrate how cultural diversity at the country level can be understood as a combination of widely shared institutions and specific cultural traditions at the individual level. Once we understand how to conceptualize the complexity of cultural components, we will study an ethnographic case to apply these ideas to patterns of social interaction (i.e., personal networks) in a multiethnic enclave, specifically the town of Sohar in Oman (Barth, 1983). By analyzing ethnographic data from Oman, we can gain insights into how patterns and compositions of personal networks reflect these cultural variations, especially regarding gender segregation. After developing our theoretical frameworks, we will present the methods and data from a survey conducted by the authors across six countries to test and interpret the results. Finally, we will outline our conclusions and the limitations of our work.”

 #3. In this sense, it is stated that “the personal network structures across countries contain traces of cultural signatures that enhance the classification of the countries of origin of egos.” However, if I understand the models presented correctly, it seems that what is being shown is that personal networks have explanatory value regarding the country of origin, even considering the variability of social norms and other demographic aspects in the sample. Is stating that these are “cultural signatures” an interpretation by the authors or a direct description of the results? In the new theoretical section, we elaborate on the social mechanisms explaining this phenomenon, and interpret the empirical results accordingly. “In sum, from our theoretical framework we would expect that people within a given country will share a set of common cultural institutions despite their internal diversity. Among these shared cultural institutions, we expect that the corporative ones (those that connect shared values, behaviours and membership) will leave identifiable traces in personal networks structures, specifically, in cohesive network measures. These patterns or “cultural signatures” should be present in the set of individual propriospects, and should contribute to predict the country of orign more accurately.

 #4. Likewise, the conclusions state that “personal network structural measures significantly contribute to predicting respondents’ cultural background, specifically the country of origin in this case.” However, isn’t the ability to predict country of origin the only thing that has been empirically verified? Yes, indeed. But the contribution of our paper in our opinion is the theoretical explanation of why this prediction happens.

 #5. After the Results section, there is a jump to the “propriospect approach” that is difficult for the reader to connect to the preceding analysis. Does this correspond to the Discussion of the previous results? Or is the intention to connect different case studies? It would be beneficial to improve the integration of the different sections that make up the text.

   Absolutely. We have rewriten the paper and changed its structure to make this transition clearer and smoother.  In particular, we added the section “Theorizing cultural signatures in personal networks” after the propriospect section and before presenting the data and methods used to test our hypotheses.

 #6. Minor corrections: In the list “that capture the individuals’ moral cosmopolitanism orientation, b a classification of cultural schemas (...)” I think adding a), b), c) would improve readability. Fixed. Thanks for pointing this out.

 #7. It seems that something is missing in this sentence: “(1) a model that only relies on the without any structural information (+170%);” Fixed as well (“sociodemographic data”).

 #6. It would be helpful to put the direct quote from Goodenough in quotation marks to indicate where the quote begins and ends. Quotation marks added.

 #8. Review: “Following Valdés and Valdés (1989)”. It seems is only Valdés. In fact, the two authors have the same surname. #8. Correct: “2025for a discussion”. Fixed the typo, thanks.

 #9. Please check if the following text in the reference list is correct: “Omani Town: Fredrik Barth, in his book Sohar. Culture and Society in an Omani Town, describes the life of a multiethnic city, seeking the mechanisms that explain the reproduction of its cultural diversity; 1983.”               Reference corrected, thank you.
